# The 'Harmonizing Optimal Strategy for Treatment of coronary artery stenosis - sAfety & effectiveneSS of drug-elUting stents & antiplatelet REgimen' (HOST-ASSURE) trial: study protocol for a randomized controlled trial

**DOI:** 10.1186/1745-6215-13-29

**Published:** 2012-03-31

**Authors:** Kyung Woo Park, Byoung-Eun Park, Si-Hyuck Kang, Jin-Joo Park, Seung-Pyo Lee, Kwang Soo Cha, Jay Young Rhew, Hui-Kyoung Jeon, Eun Seok Shin, Ju Hyeon Oh, Myung-Ho Jeong, Sanghyun Kim, Kyung-Kuk Hwang, Jung-Han Yoon, Sung Yun Lee, Tae-Ho Park, Keon Woong Moon, Hyuck-Moon Kwon, In-Ho Chae, Hyo-Soo Kim

**Affiliations:** 1 Department of Internal Medicine, Cardiovascular Center, Seoul National University Hospital, 101 Daehang-no, Seoul 110-744, South Korea; 2 Dankook University Hospital, Cheonan, South Korea; 3 Busan National University Hospital, Busan, South Korea; 4 Presbyterian Medical Center, Jeonju, South Korea; 5 Uijeongbu St. Mary's Hospital, Uijeongbu, South Korea; 6 Ulsan University Hospital, Ulsan, South Korea; 7 Samsung Changwon Hospital, Changwon, South Korea; 8 Chonnam National University Hospital, Gwangju, South Korea; 9 Boramae Medical Center, Seoul, South Korea; 10 Chungbuk National University Hospital, Cheongju, South Korea; 11 Wonju Christian Hospital, Wonju, South Korea; 12 Inje University Ilsan Paik Hospital, Goyang, South Korea; 13 Dong-A Medical Center, Busan, South Korea; 14 St. Vincent's Hospital, Suwon, South Korea; 15 Gangnam Severance Hospital, Seoul, South Korea; 16 Seoul National University Bundang Hospital, Seongnam, South Korea

**Keywords:** Cilostazol, cobalt chromium, coronary heart disease, everolimus-eluting stent, platinum chromium, zotarolimus-eluting stent

## Abstract

**Background:**

Second-generation drug-eluting stents (DES) have raised the bar of clinical performance. These stents are mostly made from cobalt chromium alloy. A newer generation DES has been developed from platinum chromium alloy, but clinical data regarding the efficacy and safety of the platinum chromium-based everolimus-eluting stent (PtCr-EES) is limited, with no comparison data against the cobalt chromium-based zotarolimus-eluting stent (CoCr-ZES). In addition, an antiplatelet regimen is an integral component of medical therapy after percutaneous coronary intervention (PCI). A 1-week duration of doubling the dose of clopidogrel (double-dose antiplatelet therapy (DDAT)) was shown to improve outcome at 1 month compared with conventional dose in acute coronary syndrome (ACS) patients undergoing PCI. However in Asia, including Korea, the addition of cilostazol (triplet antiplatelet therapy (TAT)) is used more commonly than doubling the dose of clopidogrel in high-risk patients.

**Methods:**

In the 'Harmonizing Optimal Strategy for Treatment of coronary artery stenosis - sAfety & effectiveneSS of drug-elUting stents & antiplatelet REgimen' (HOST-ASSURE) trial, approximately 3,750 patients are being prospectively and randomly assigned in a 2 × 2 factorial design according to the type of stent (PtCr-EES vs CoCr-ZES) and antiplatelet regimen (TAT vs DDAT). The first primary endpoint is target lesion failure at 1 year for the stent comparison, and the second primary endpoint is net clinical outcome at 1 month for comparison of antiplatelet therapy regimen.

**Discussion:**

The HOST-ASSURE trial is the largest study yet performed to directly compare the efficacy and safety of the PtCr-EES versus CoCr-ZES in an 'all-comers' population. In addition, this study will also compare the clinical outcome of TAT versus DDAT for 1-month post PCI.

**Trial registration:**

ClincalTrials.gov number NCT01267734.

## Background

Various second-generation drug-eluting stents (DES) have been developed in the hopes of improving both efficacy and safety compared with first-generation DES. Cumulative evidence is building suggesting that compared with the paclitaxel-eluting stent, the second-generation everolimus-eluting stent (EES; Xience V, Abbott Vascular, Santa Clara, CA, USA, Promus, Boston Scientific, Natick, MA, USA) is more effective and safe [1-3], and compared with the sirolimus-eluting stent, it has similar efficacy with a trend toward better safety [4-6]. The next addition to the second-generation DES market has been the zotarolimus-eluting stent (ZES; Resolute, Medtronic, Minneapolis, MN, USA), which showed equivalent outcome compared with the EES in the RESOLUTE All-Comers randomized trial [7]. Both EES and ZES are based on a cobalt chromium alloy stent. Most recently, a newly developed stent alloy, the platinum chromium alloy, was incorporated with everolimus to create the platinum chromium-based EES (PtCr-EES), the Promus Element stent (Boston Scientific, Natick, MA, USA). There are limited data on the clinical efficacy and safety of the PtCr-EES with only one modestly sized randomized trial that compared it against the cobalt chromium-based EES in a select group of patients and lesions [8]. In the current study, the PtCr-EES will be compared against the cobalt chromium-based ZES (CoCr-ZES) in a larger scale study with a broader all-comers population.

In addition, antiplatelet regimen is an integral component of medical therapy after percutaneous coronary intervention (PCI). In particular, inhibition of platelet reactivity in the first month post PCI is known to be critical in preventing thrombotic events, since higher on-treatment platelet reactivity is reported to be associated with higher risk of hard endpoints [9-12]. In the CURRENT-OASIS 7 trial, a 1-week duration of doubling the dose of clopidogrel was shown to improve outcome at 1 month compared with conventional dose in acute coronary syndrome (ACS) patients undergoing PCI [13]. In Asia including Korea, the addition of cilostazol as a third antiplatelet agent is used more commonly than doubling the dose of clopidogrel in high-risk patients. However, there has been no large-scale comparison of triple antiplatelet therapy (TAT) versus double-dose clopidogrel antiplatelet therapy (DDAT) to date.

## Methods

### Study objectives and hypotheses

The primary objective of the present study is to compare the efficacy and safety of coronary stenting with the PtCr-EES compared with the CoCr-ZES in the treatment of coronary stenosis, and to evaluate the efficacy and safety of a 1 month duration of TAT consisting of aspirin, clopidogrel 75 mg, and cilostazol 200 mg versus DDAT consisting of aspirin and clopidogrel 150 mg. The working hypothesis of this trial is that PtCr-EES is non-inferior to CoCr-ZES in prevention of target lesion failure (TLF) at 1-year post PCI, and TAT is non-inferior to DDAT regarding net clinical outcome, defined as a composite of cardiac death, non-fatal myocardial infarction (MI), stent thrombosis, stroke and PLATO ('PLATelet inhibition and patient Outcomes') major bleeding at 1 month [14].

### Study design

**Figure 1:**
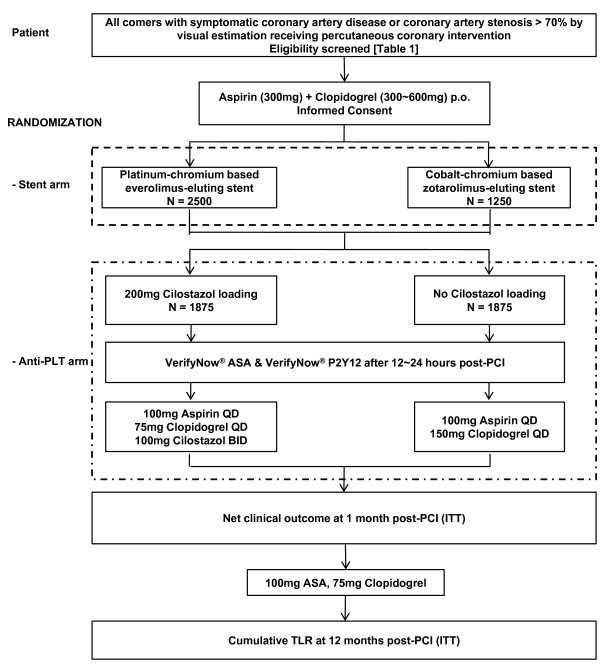
**Study algorithm**.

This is a prospective, randomized, single blind, blinded endpoint evaluation, multicenter trial with a 2 × 2 factorial design. The study algorithm is shown in Figure 1. After enrollment and index PCI procedure, clinical follow-up will occur at 1, 3, 12 months and yearly up to 3 years after intervention. Follow-ups will be conducted as telephone contacts or office visits. Unless clinically necessary, there will be no angiographic follow-up until 12 months post PCI, the timepoint for the primary endpoint of the stent comparison. In a subset of patients angiographic follow-up will be recommended at 13 months post PCI. In another subgroup of patients, clopidogrel on-treatment platelet reactivity will be measured using the VerifyNow P2Y12 assay at baseline PCI and at 1-month follow-up. This study is an investigator-initiated clinical trial with grant support from two sources, the Ministry of Health, Welfare, and Family Affairs of the Republic of Korea, and Boston Scientific Korea. Other than providing financial support, Boston Scientific was not involved with the protocol development or the study process, including site selection, management, and data collection and analysis. The authors are solely responsible for the design and conduct of this study, all study analyses, the drafting and editing of the paper and its final contents.

### Study population

Patients that are at least 18 years of age who show on coronary angiogram to have significant coronary artery or graft stenosis and meet all of the inclusion and exclusion criteria (Additional file 1: Table S1) are eligible for this study. This will be an 'all-comers' study, with no angiographic exclusion criteria in order to mimic real world clinical practice and will include the likes of multivessel stenting, left main stenting, chronic total occlusion intervention, bifurcation stenting, saphenous vein graft stenting, previous stent restenosis, and so on. The major exclusion criteria for study enrollment will be known hypersensitivity or contraindication to heparin, aspirin, clopidogrel, cilostazol, everolimus, zotarolimus, or contrast media, systemic (intravenous) us of everolimus or zotarolimus within 12 months, females of childbearing potential, unless a recent pregnancy test is negative, a history of bleeding diathesis, known coagulopathy (including heparin-induced thrombocytopenia), abnormal hemogram (hemoglobin < 10 g/dl or platelet count < 100,000 cells/μl) or will refuse blood transfusions, patients with severe left ventricular systolic dysfunction (left ventricular ejection fraction < 25%), those with cardiogenic shock, gastrointestinal or genitourinary bleeding within the prior 3 months, or major surgery within 2 months, and symptomatic heart failure. The patients that agree to participate in the study and give written informed consent, will be randomized 2:1 to either (a) PtCr-EES or (b) CoCr-ZES, and 1:1 to either (a) TAT or (b) DDAT (2 × 2 design). A total of 3,750 patients plan to be enrolled from 40 high-volume hospitals in Korea, the names and investigators of which are listed in Additional file 2: Appendix A.

### Interventions and study device, drug

PCI will be performed with the standard technique and device, that is, catheter, guidewire and balloon catheter. The decision to predilate the lesion, use glycoprotein IIb/IIIa inhibitors and dilate the stent adjunctively will be up to the operator's discretion. The Promus Element stent is available in diameters of 2.25, 2.5, 2.75, 3.0, 3.5 and 4.0 with lengths of 8, 12, 16, 20, 24, 28, 32, and 38 mm (32 and 38 mm lengths not available for 2.25 and 2.5 mm diameters). The Endeavor Resolute stent is available in diameters of 2.5, 2.75, 3.0, 3.5 and 4.0, with lengths of 12, 15, 18, 24, 30 and 38 mm (except for 2.5 mm stents which are available as 14 mm length instead of 15 mm, and 38 mm lengths not available for 2.5 and 2.75 mm diameters). It will be recommended that all diseased coronary or graft lesions that are the targets of intervention be fully covered by the stent, with non-diseased tissue at each end of the stent. In cases of long lesions that cannot be fully covered by one stent, stent overlap of approximately 1 mm to 4 mm will be recommended per manufacturer's instruction for use (IFU). It will be recommended that the allocated stent be implanted in all lesions treated in the same patient. However, other stents may be used in case of device failure, or situations where the operator decides that it is in the best interest of the patient to do so. The use of intravascular ultrasound (IVUS) or optical coherence tomography (OCT) to guide in the procedure will also be up to the operator's discretion.

All patients will receive approximately 300 to 600 mg of clopidogrel and 300 mg of aspirin loading before the procedure. The patients randomized to the TAT group will receive an additional loading of 200 mg cilostazol (Otsuka, Korea) followed by 100 mg twice a day maintenance. Those randomized to the DDAT group will receive 150 mg of clopidogrel maintenance.

### Outcome measures

There are two primary endpoints in this study. The primary endpoint of the trial for the stent comparison will be TLF, defined as a composite of cardiac death, target vessel-related non-fatal MI and ischemia-driven target lesion revascularization (TLR) at 12 months. The primary endpoint for the antiplatelet regimen comparison will be net clinical outcome, defined as a composite of cardiac death, non-fatal MI, stent thrombosis, stroke and PLATO major bleeding at 1 month [14]. The individual component definitions appear in Additional file 2: Appendix B. Stent thrombosis will be adjudicated according to the definitions of the Academic Research Consortium [15]. All endpoints will be analyzed principally on an intention-to-treat basis.

Secondary endpoints of the study will include all of the individual components of the primary endpoint. In addition, all death, ischemia-driven target vessel revascularization (TVR), target vessel failure (TVF), acute success (device, procedure) will be included as secondary endpoints. In the subgroup of patients that receive 13-month angiographic follow-up, both stents will also be analyzed using quantitative coronary angiography (QCA) for late luminal loss, percentage diameter stenosis, IVUS for percentage neointimal volume, or OCT for percentage neointimal coverage. In another subgroup of patients with on-treatment platelet reactivity (OPR) measurement, OPR will be compared between the two antiplatelet regimens, and according to genotype status. The effect of OPR on clinical outcome will also be analyzed.

### Statistical considerations

#### Sample size calculations

To test the first hypothesis that the PtCr-EES is non-inferior to CoCr-ZES in reducing 12-month cumulative TLF after PCI, we assumed the TLF rate of PtCr-EES and CoCr-ZES to be 6.5%, based on the results from previous randomized trials [1,2,7]. Using a non-inferiority log-rank design with a non-inferiority margin of hazard ratio 1.5, sampling ratio of EES:ZES at 2:1, allowing 5% attrition rate for the 12 months, a total of 3,750 patients would result in a power of at least 80% power with an one-sided α of 2.5%.

Also, to test the second hypothesis that TAT is non-inferior to DDAT regarding 1-month net clinical outcome, we assumed the net clinical outcome rate of TAT and DDAT to be 2.0% and 3.0%, respectively. The net clinical outcome is defined as a composite of cardiac death, non-fatal MI, stent thrombosis, stroke and PLATO major bleeding at 1 month. With a non-inferiority design, a non-inferiority margin of 0.75%, 1:1 randomization to either groups and allowing for 2.5% attrition for 1-month clinical follow-up, a total of 3,750 patients would provide a one-sided α of 2.5% and > 90% power.

### Statistical analyses

All primary and secondary endpoints will be analyzed on an intention-to-treat basis (all patients analyzed as part of their assigned treatment group). The endpoints will also be analyzed both on a per-patient and per-lesion basis, whenever applicable. For patients receiving multivessel PCI, the index lesion analyzed in the per-patient analysis will be determined randomly by a computer prior to QCA analysis. For intent-to-treat analysis, all patients who signed the written informed consent form and are randomized in the study will be included in the analysis sample, regardless of whether or not the correct treatment was administered, or whether crossover occurred.

The second primary endpoint of 12-month cumulative TLF and 1-month cumulative net clinical outcome will be analyzed by comparing the Kaplan-Meier event rates using the log rank test on an intention-to-treat basis. The null hypothesis will be evaluated using a non-inferiority statistics. The outcomes will also be analyzed according to subgroups defined a priori. These include the following: for comparison of the stent arm, subgroup analysis will be performed according to the lesion length, vessel size, renal dysfunction, multivessel stenting, and presence or absence of diabetes mellitus; for comparison of the antiplatelet arm, subgroup analysis will be performed according to the prescription status of calcium-channel blockers, 3-hydroxy-3-methylglutaryl-coenzyme A (HMG-CoA) reductase inhibitors, allocated stent, renal dysfunction, multivessel stenting, and the presence or absence of diabetes mellitus. The multiple imputation method will be used to handle missing data.

### Trial organization and ethical considerations

The overall trial organization is summarized in Additional file 2: Appendix C. The trial was designed by the principal investigators and the executive committee. Besides the executive committee, the steering committee, data safety monitoring board, and the clinical event adjudication committee will be involved in the execution, administration, and supervision of the trial. The specific role and information regarding each of the committees appear in Additional file 2: Appendix C. The study will be sponsored by the Seoul National University Hospital (SNUH) Cardiovascular Clinical Research Center (CCRC) and the data will be managed by an independent contract research organization, Dream CIS Inc. Dream CIS will be responsible for the development of a web-based randomization system, maintenance of the web-based case report form, and data collection from individual sites. Site monitoring will be performed by the CCRC of SNUH. The trial monitors will review the documents of at least 30% of the patients enrolled from each participating center, at appropriate intervals, for accuracy and completeness and to ensure compliance with the protocol. The trial monitor will be able to inspect all documents and required records that are maintained by the individual investigator and site, including medical records (office, clinic, or hospital) for the subjects in this trial. The study will be performed in accordance with the Declaration of Helsinki. Before participation of the study, each patient will be given full information, that is, purpose, methods, rights, duties and possible risk/benefits of the study in plain, lay language. Written, informed consent is a prerequisite to the participation in the study.

### Ethical approval

This study was approved by the institutional review board at Seoul National University Hospital (D-1005-001-068) and by the local ethical committee at each participating hospital. The protocol of the trial has been registered at http://www.clinicaltrials.gov (NCT01267734).

## Discussion

### EES and ZES in clinical practice

The second-generation CoCr-EES (Xience V, Promus) has been shown to be either comparable or better than its first-generation counterparts. In large scale randomized trials against paclitaxel-eluting stents (PES), such as the SPIRIT IV and COMPARE trials, it was superior to PES with regard to both efficacy and safety where the primary clinical endpoints were reduced by 38% and 31%, respectively [1,2], which has now been reaffirmed in a meta-analysis [16]. In randomized trials and meta-analysis comparing CoCr-EES and SES, such as the EXCELLENT, ISAR-TEST 4, SORT-OUT IV, and so on, CoCr-EES showed similar efficacy with slight trend toward superior safety [4-6]. The next addition to the second-generation DES market has been the CoCr-ZES (Endeavor Resolute), which showed equivalent outcome compared with the CoCr-EES in the RESOLUTE All-Comers randomized trial [7]. Because of the all-comers population that was studied in this trial, data were available regarding to the performance of the second-generation stents in complex lesions such as coronary total occlusions, bifurcation lesions, saphenous vein grafts, in-stent restenosis, unprotected left main lesions, multivessel stenting, and so on, which constituted approximately two-thirds of the lesions treated in the trial [17]. In this complex subgroup, there were no significant differences between the two DES devices but the TLF rate at 1 year numerically favored CoCr-ZES. Both of these second-generation DES are based on a cobalt chromium alloy stent. Most recently, a newly developed stent alloy, the platinum chromium alloy, was incorporated with everolimus to create the PtCr-EES, the Promus Element stent (Boston Scientific). In the only randomized trial to date with the PtCr-EES, the PLATINUM trial, where it was compared against CoCr-EES in a select group of patients with various angiographic exclusions [8], PtCr-EES was non-inferior to CoCr-EES for the primary endpoint of TLF, with insignificant differences in measures of safety and efficacy through 1 year. Both stents showed low event rates at 1 year (TLF rate: 3.4% vs 2.9% for PtCr-EES vs CoCr-EES, *P *= 0.60). However the PtCr-EES has not been studied randomly in a broader population of patients as was enrolled in the RESOLUTE All-Comers trial, which better reflects the day-to-day clinical practice in most centers of coronary intervention. The non-inferiority margin of 3.25% may seem large given that the estimated rate of the primary endpoint is 6.5%. However, in the RESOLUTE All-Comers trial and the PLATINUM trial, well known previous studies incorporating the non-inferiority design regarding stent comparisons, a non-inferiority margin of 3.5% has been used [7,8]. In addition, because of the paucity of the data on PtCr-EES, the sampling ratio was set at 2:1 to maximize the number of patients randomized to the PtCr-EES arm.

### Antiplatelet regimen after PCI

Antiplatelet therapy is one of the most important components of successful outcome after PCI. High on-treatment platelet reactivity (HOPR) has been shown to be an independent predictor of thrombotic outcomes [9-12]. In particular, the first month after PCI is the most important period to inhibit platelet function to prevent thrombotic events, since the greatest association between heightened platelet reactivity and thrombotic outcome is seen in the first month post PCI [18]. In Korea, where the HOPR rate of the general population receiving PCI is above 50% and frequency of the cytochrome P450 2C19 (CYP2C19) loss-of-function (LOF) allele is greater than 60% [12,19-21], we may need increased platelet inhibition during this critical period.

To increase platelet inhibition after PCI, the approach is slightly different between Western and Asian doctors. Before prasugrel and ticagrelor, most Western doctors prescribed double dose of clopidogrel in high-risk settings. However, in Asia and in particular in Korea, doctors prefer addition of cilostazol on conventional dual antiplatelet therapy (DAPT) to doubling the dose of clopidogrel. In the CURRENT-OASIS 7 trial, a 1-week duration of doubling the dose of clopidogrel was shown to improve outcome at 1 month compared with conventional dose in ACS patients undergoing PCI [13]. The basis of adding cilostazol in Korea comes from pharmacodynamic studies from our group and others, which have shown that TAT is superior to DDAT with regard to inhibition of platelet reactivity in patients with high platelet reactivity [22], and in carriers of the CYP2C19 LOF allele [23]. In the DECLARE-Diabetes, and DECLARE-Long studies, TAT was superior to conventional DAPT regarding inhibition of neointima formation and significantly reduced rates of clinically driven TLR [24,25]. In the CILON-T trial, which was underpowered to show differences in clinical outcome, there were no significant difference between TAT and conventional DAPT although TAT more profoundly reduced OPR [26]. However in the *post hoc *analysis, we showed that there was a significant trend toward worse outcome in those with high OPR suggesting that cilostazol may have some beneficial effects in higher-risk populations.

Because of the paucity of sound clinical data regarding the use of TAT based on randomized clinical trials, and in light of the abundant use of cilostazol as a third agent in post-PCI management in Korea, a large scale randomized controlled trial (RCT) to confirm the safety and efficacy of TAT is needed at present. After initiation of the 'Harmonizing Optimal Strategy for Treatment of coronary artery stenosis - sAfety & effectiveneSS of drug-elUting stents & antiplatelet REgimen' (HOST-ASSURE) trial, we anticipate enrollment within 1 year. The large scale and all-comers design of the HOST-ASSURE trial will provide important insight into two issues in interventional cardiology. First, it will test whether the newest DES platform, PtCr-EES will be as effective and safe as CoCr-ZES in preventing TLF. Second, it will also address the issue of whether TAT is non-inferior to DDAT in reducing net clinical outcome within the first month post PCI.

### Trial status

The HOST-ASSURE Trial is ongoing. Patient recruitment has been completed and follow-up is being conducted.

## Competing interests

The authors declare that they have no competing interests.

## Authors' contributions

KWP and B-EP contributed to the initial conception, study design, data, acquisition, and wrote the manuscript, S-HK, J-JP, and S-PL contributed to the study design, data acquisition, data interpretation, and drafting of the manuscript. KSC, JYR, H-KJ, ESS, and JHO contributed to patient enrollment, critical reading and revision of the manuscript. M-HJ, SK, K-KH, and J-HY contributed to the study design, and drafting of the protocol. SYL, T-HP, KWM, and H-MK contributed to patient enrollment, data acquisition, and critical reading and revision of the manuscript. I-HC and H-SK contributed to the initial conception of the study, protocol development, writing of the manuscript and final approval of the submitted version. KWP and B-EP contributed equally. All authors read and approved the final manuscript.

## Supplementary Material

Additional file 1 **Table S1: Enrollment criteria**. Click here for file

Additional file 2 **Appendices A-C**. Appendix A: participating high-volume hospitals in Korea. Appendix B: endpoint definitions. Bleeding/hemorrhagic complications. Appendix C: trial organization. Click here for file
