# Supplementary material for: The 'Harmonizing Optimal Strategy for Treatment of coronary artery stenosis - sAfety & effectiveneSS of drug-elUting stents & antiplatelet REgimen' (HOST-ASSURE) trial: study protocol for a randomized controlled trial
Source: Trials. 2012 Mar 31;13:29. doi: 10.1186/1745-6215-13-29 (PMC3342216; doi:10.1186/1745-6215-13-29)
Supplement: Additional file 1 — Table S1: Enrollment criteria. [file 1745-6215-13-29-S1.DOC]

**Appendix A. Participating high-volume hospitals in Korea**

| **No** | **Hospital Name** | **Principal Investigator** |
| --- | --- | --- |
|
| 1 | Seoul National University Hospital | Kim, Hyo-Soo |
| 2 | Seoul National University Bundang Hospital | Chae, In-Ho |
| 3 | Pusan National University Hospital | Cha, Kwang Soo |
| 4 | Dankook University Hospital | Park, Byoung Eun |
| 5 | Presbyterian Medical Center | Rhew, Jay Young |
| 6 | Uijeongbu St. Mary’s Hospital | Jeon, Hui-Kyung |
| 7 | Ulsan University Hospital | Shin, Eun Seok |
| 8 | Samsung Changwon Hospital | Oh, Ju Hyeon |
| 9 | Chonnam National University Hospital | Jeong, Myung-Ho |
| 10 | Chungbuk National University Hospital | Hwang, Kyung-Kuk |
| 11 | Wonju Christian Hospital | Yoon, Jung-Han |
| 12 | Inje University Ilsan Paik Hospital | Lee, Sung Yun |
| 13 | Boramae Medical Center | Kim, Sanghyun |
| 14 | Dong-A Medical Center | Park, Tae-Ho |
| 15 | Gangnam Severance Hospital | Kwon, Hyuck-Moon |
| 16 | St. Vincent’s Hospital | Moon, Keon Woong |
| 17 | Daegu Catholic University Medical Center | Ryu, Jae-Kean |
| 18 | Keimyung University Dongsan Medical Center | Hur, Seung-Ho |
| 19 | Daegu Fatima Hospital | Lee, Bong-Ryul |
| 20 | Gyeongsang National University Hospital | Park, Yong-Whi |
| 21 | Konyang University Hospital | Bae, Jang-Ho |
| 22 | Hallym University Kangdong Sacred Heart Hospital | Han, Kyoo-Rok |
| 23 | Ewha Womans University Mokdong Hospital | Park, Si-Hoon |
| 24 | Korea University Guro Hospital | Rha, Seung-Woon |
| 25 | Hallym University Sacred heart Hospital | Park, Woo-Jung |
| 26 | Wongwang University Hospital | Oh, Seok-Kyu |
| 27 | Korea University Anam Hospital | Lim, Do-Sun |
| 28 | Kwangju Christian Hospital | Lee, Seung-Wook |
| 29 | Hallym University Chuncheon Sacred Heart Hospital | Yoon, Duck-Hyoung |
| 30 | Kyung Hee University Hospital at Gangdong | Kim, Chong-Jin |
| 31 | Seoul Medical Center | Kim, Seok-Yeon |
| 32 | Gachon University Gil Hospital | Ahn, Taehoon |
| 33 | Samsung Medical Center | Gwon, Hyeon-Cheol |
| 34 | Hallym University Kangnam Sacred Heart Hospital | Lee, Namho |
| 35 | National Health Insurance Medical Center | Jeon, Dong-Woon |
| 36 | Soonchunhyang University Hospital | Hyun, Min-Soo |
| 37 | Daejun Eulji University Hospital | Lee, Sang |
| 38 | Hanyang University Guri Hospital | Lee, Jaewoong |
| 39 | Kangwon National University Hospital | Ryu, Dong Ryeol |
| 40 | Kosin University Gospel Hospital | Cha, Tae-Joon |

**Appendix B. End point definitions**

**Bleeding/Hemorrhagic Complications**

An episode of bleeding is defined by the PLATO criteria as:

| Term |  | Associated decrease in hemoglobin | Transfusion of whole blood or PRBCs for bleeding |
| --- | --- | --- | --- |
| **Major bleed**—life threatening; meets any of these criteria: | Fatal or intracranial or intrapericardial with cardiac tamponade or hypovolemic shock or severe hypotension requiring pressors or surgery | >50 g/L  (3.1 mmol/L) | ≥4 U |
| **Major bleed**—other; meets any of these criteria: | Significantly disabling (eg, intraocular with permanent vision loss) | 30-50 g/L  (1.9-3.1 mmol/L) | 2-3 U |
| **Minor bleed** | Requires medical intervention to stop or treat bleeding |  |  |
| **Minimal bleed** | All others not requiring intervention or treatment |  |  |

*To account for transfusion, Hgb and Hct measurements will be adjusted for any packed red blood cells or whole blood given between baseline and post-transfusion measurements. A transfusion of one unit of blood will be assumed to result in an increase of 1 g/dL in Hgb or of 3% in Hct. Thus, to calculate the true change in Hgb or Hct if there has been an intervening transfusion between two blood measurements, the following calculations should be performed:

Hgb = [baseline Hgb . post transfusion Hgb] + [number of transfused units]

Hct. = [baseline Hct . post transfusion Hct] + [number of transfused units x 3]

An episode of bleeding is defined by the TIMI criteria as:

1. Major: Overt clinical bleeding (or documented intracranial or retroperitoneal hemorrhage) associated with a drop in hemoglobin of greater than 5 g/dl (0.5 g/l) or in hematocrit of greater than 15% (absolute) **Note: A patient who experiences an intracranial hemorrhage should be considered to have a major hemorrhage.*
2. Minor: Overt clinical bleeding associated with a fall in hemoglobin of 3 to less than or equal to 5 g/dl (0.5 g/l) or in hematocrit of 9% to less than or equal to 15% (absolute)
3. None: No bleeding event that meets the major or minor definition **Note: In calculating the fall in hemoglobin or hematocrit, a transfusion of whole blood or packed red blood cells is counted as 1 g/dl (0.1 g/l) hemoglobin or 3% absolute in hematocrit. This would be in addition to the actual fall in hemoglobin or hematocrit.*

*To account for transfusion, Hgb and Hct measurements will be adjusted for any packed red blood cells or whole blood given between baseline and post-transfusion measurements. A transfusion of one unit of blood will be assumed to result in an increase of 1 g/dL in Hgb or of 3% in Hct. Thus, to calculate the true change in Hgb or Hct if there has been an intervening transfusion between two blood measurements, the following calculations should be performed:

Hgb = [baseline Hgb . post transfusion Hgb] + [number of transfused units]

Hct. = [baseline Hct . post transfusion Hct] + [number of transfused units x 3]

The following will be classified as “Instrumented” Major Bleeding that is considered to be associated with the catheterization laboratory visit:

1. Major Percutaneous Entry Site: Bleeding occurred at the percutaneous entry site during or after the catheterization laboratory visit until discharge. The bleeding should require a transfusion and/or prolong the health care facility stay, and/or cause a drop in Hgb > 5 g/dL. Bleeding at the percutaneous entry site can be external or a hematoma >10 cm for femoral access or > 2 cm for radial access; or > 5 cm for brachial access.

2. Major Retroperitoneal, Gastrointestinal, and Genital/Urinary: Bleeding occurred during or after the catheterization laboratory visit until discharge. The bleeding either requires surgical intervention (eg, to relieve nerve compression), and/or requires a transfusion and/or prolong the health care facility stay, and/or cause a drop in hemoglobin > 5.0 g/dL.

3. Major Other/Unknown: Bleeding occurred at other or unknown locations during or after the catheterization laboratory visit until discharge. The bleeding should require a transfusion and/or prolong the health care facility stay, and/or cause a drop in Hgb > 5 g/dL.

**Cardiac death**

Death due to acute MI; cardiac perforation/pericardial tamponade; arrhythmia or conduction abnormality; cerebrovascular accident within 30 days of the procedure or cerebrovascular accident suspected of being related to the procedure; and death due to complications of the procedure, including bleeding, vascular repair, transfusion reaction, or bypass surgery; or any death in which a cardiac cause cannot be excluded.

**Net clinical outcome**

A composite of cardiac death, nonfatal MI, stent thrombosis, CVA and major bleeding (defined by the PLATO criteria) at 1 month

**Stent thrombosis**

Definite stent thrombosis is considered to have occurred by either angiographic or pathological confirmation.

- Angiographic confirmation of stent thrombosis:  The presence of a thrombus that originates in the stent or in the segment 5 mm proximal or distal to the stent and presence of at least 1 of the following criteria within a 48-hour time window:
- Pathological confirmation of stent thrombosis: Evidence of recent thrombus within the stent determined at autopsy or via examination of tissue retrieved following thrombectomy.

Clinical definition of probable stent thrombosis is considered to have occurred after intracoronary stenting in the following cases:

- Any unexplained death within the first 30 days,
- Irrespective of the time after the index procedure, any MI that is related to documented acute ischemia in the territory of the implanted stent without angiographic confirmation of stent thrombosis and in the absence of any other obvious cause

Clinical definition of possible stent thrombosis is considered to have occurred with any unexplained death from 30 days after intracoronary stenting until end of trial follow-up.

Timing of stent thrombosis:

- Acute stent thrombosis: 0 to 24 hours after stent implantation
- Subacute stent thrombosis: >24 hours to 30 days after stent implantation
- Late stent thrombosis: >30 days to 1 year after stent implantation
- Very late stent thrombosis: >1 year after stent implantation

**Stroke [=Cerebrovascular accident (CVA)]**

A stroke is defined as a focal loss of neurological function caused by an ischemic or hemorrhagic event with residual symptoms at least 24 hours after onset or leading to death.

Sudden onset of vertigo, numbness, aphasia, dysarthria or central neurologic deficit secondary to vascular lesions of the brain such as hemorrhage, embolism, thrombosis, or rupturing aneurysm with residual symptoms at least 24 hours after onset or leading to death.

* CVA type

1. Hemorrhagic: A stroke with documentation on imaging (e.g., CT scan or MRI of hemorrhage in the cerebral parenchyma, or a subdural or subarachnoid hemorrhage). Evidence of hemorrhagic stroke obtained from lumbar puncture, neurosurgery, or autopsy can also confirm the diagnosis.
2. Nonhemorrhagic: A focal neurological deficit that results from a thrombus or embolus (and not due to hemorrhage) that appears and is still partially evident for more than 24 hours
3. Unknown/no imaging performed: if the type of stroke could not be determined by imaging or other means (from lumbar puncture, neurosurgery, or autopsy)

**Target lesion failure**

A composite of cardiac death, target vessel-related nonfatal myocardial infarction (MI) and ischemia-driven target lesion revascularization (TLR) at 12 months

**Target lesion revascularization (Ischemia-driven)**

Repeat percutaneous intervention of the target lesion (including 5-mm margins on both edges of a stent) or bypass surgery of the target vessel associated with 1 of the following: (1) positive functional ischemia study; (2) ischemic symptoms and angiographic minimal lumen diameter stenosis ≥50% by core laboratory quantitative coronary angiography (QCA); or (3) angiographic diameter stenosis ≥70% by core laboratory QCA without either ischemic symptoms or a positive functional study.

**Target vessel-related nonfatal Myocardial infarction**

An elevation of CK to ≥3 times the upper limit of normal with elevated CK-MB in the absence or presence of new pathological Q waves on the electrocardiogram (non–Q- and Q-wave MI, respectively). The basis for evaluating whether MI is related to the target vessel will be the presence of electrocardiographic signs of acute ischemia in the territory of the target vessel and/or on the index event angiogram, if available. All MIs that cannot be clearly attributed to a vessel other than the target vessel will be considered related to the target vessel.

**Appendix C. Trial organization**

1. Executive Committee

The Executive Committee will be composed of the study Chairperson and selected members among the investigators. This committee is responsible for overseeing the administrative progress of the study and will approve the final trial design and protocol issued to the Data and Safety Monitoring Board (DSMB) and the clinical sites. This committee will also be responsible for reviewing the final results, determining the methods of presentation and publication, and selection of secondary projects and publications by members of the Steering Committee. The executive committee also holds the right to modify or stop the study prematurely based on recommendations from the DSMB.

2. Steering Committee

The Steering Committee will be composed of the principal investigators from the centers participating in this trial. The committee is responsible for the day-to-day administrative management of the trial and will meet on a regular base to monitor subject enrollment, clinical site progress, and protocol compliance. It will be the responsibility of the steering committee to provide assistance and education to individual sites and researchers to help with trial management, record keeping, and reporting requirements. The steering committee will prepare reports to be reviewed by the Executive Committee.

3. Data Safety Monitoring Board (DSMB)

The DSMB will be composed of general and interventional cardiologists, and a biostatistician. Names of the actual members will not be announced, but may be provided to the regulatory agency upon request. The DSMB will function in accordance with applicable regulatory guidelines. The board members are independent and will not be participating in the trial. The DSMB committee will review the safety data from this study and make recommendations based on safety analyses of unanticipated device effects (UADEs), serious adverse events (SAEs), protocol deviation, device failures, and 30-day follow-up reports. The frequency of the DSMB meetings will be predetermined. Additionally, the DSMB may call a meeting at any time if there is reason to suspect that safety is an issue. The DSMB is responsible for making recommendations regarding any safety or compliance issues throughout the course of the study and may recommend to the Executive Committee to modify or stop the study. However, all final decisions regarding study modifications rest with the Executive Committee. All cumulative safety data will be reported to the DSMB and reviewed on an ongoing basis throughout enrollment and follow-up periods to ensure patient safety. Every effort will be made to allow the DSMB to conduct an unbiased review of patient safety information. All DSMB reports will be made available to the appropriate agencies upon request but will otherwise remain strictly confidential.

Prior to the DSMB’s first review of the data, the DSMB charter will be drafted. The DSMB will develop a consensus understanding of all trial endpoints and definitions used in the event adjudication process. All DSMB reports will remain strictly confidential, but will be made available to the regulatory body upon request.

4. Clinical Event Adjudication Committee (CEAC)

The Clinical Events Adjudication Committee (CEAC) is comprised of interventional and non-interventional cardiologists who are not investigators in the study. The CEAC is charged with the development of specific criteria used for the categorization of clinical events and clinical endpoints in the study which are based on protocol. All members of the CEAC will be blinded to the primary results of the trial. The CEAC will review and adjudicate all clinical events. The Committee will also review and rule on all deaths that occur throughout the trial.

5. Data Coordination and Site Management

Data coordination and site management services will be performed at the DreamCIS Incorporated and Cardiovascular Clinical Research Center of Seoul National University Hospital.
