# Supplementary material for: The 'Harmonizing Optimal Strategy for Treatment of coronary artery stenosis - sAfety & effectiveneSS of drug-elUting stents & antiplatelet REgimen' (HOST-ASSURE) trial: study protocol for a randomized controlled trial
Source: Trials. 2012 Mar 31;13:29. doi: 10.1186/1745-6215-13-29 (PMC3342216; doi:10.1186/1745-6215-13-29)
Supplement: Additional file 2 — Appendices A-C. Appendix A: participating high-volume hospitals in Korea. Appendix B: endpoint definitions. Bleeding/hemorrhagic complications. Appendix C: trial organization. [file 1745-6215-13-29-S2.DOCX]

**Table S1** Enrollment criteria

| **General inclusion criteria**  Subject must be at least 18 years of age.  Subject is able to verbally confirm understandings of risks, benefits and treatment alternatives of receiving the Promus Element or Endeavor Resolute stents, and he/she or his/her legally authorized representative provides written informed consent prior to any study related procedure.  Subject must have significant lesion (>50% by visual estimate) in any of the coronary arteries, venous or arterial bypass grafts.  Subject must have evidence of myocardial ischemia (for example, stable, unstable angina, recent infarction, silent ischemia, positive functional study or a reversible changes in the electrocardiogram (ECG) consistent with ischemia). In subjects with diameter stenosis >70%, evidence of myocardial ischemia does not have to be documented. |
| --- |
| **Angiographic inclusion criteria**  Target lesion(s) must be located in coronary artery, venous or arterial bypass graft with diameter of ≥ 2.5 mm and ≤ 4.25 mm.  Target lesion(s) must be amenable for percutaneous coronary intervention. |
| **Exclusion criteria**  The patient has a known hypersensitivity or contraindication to any of the following medications: Heparin, Aspirin, Clopidogrel, Cilostazol, Everolimus, Zotarolimus, contrast media. (Patients with documented sensitivity to contrast media that can be effectively premedicated with steroids and diphenhydramine, for example, rash, may be enrolled. Those with true anaphylaxis to prior contrast media, however, should not be enrolled.)  Systemic (intravenous) Everolimus or Zotarolimus use within 12 months.  Female of childbearing potential, unless a recent pregnancy test is negative, who possibly plan to become pregnant any time after enrollment into this study.  History of bleeding diathesis, known coagulopathy (including heparin-induced thrombocytopenia), abnormal hemogram (hemoglobin <10 g/dl or platelet count <100,000 cells/μl) or will refuse blood transfusions.  Patients with severe left ventricular systolic dysfunction (left ventricular ejection fraction <25%) or cardiogenic shock.  Gastrointestinal or genitourinary bleeding within the prior 3 months, or major surgery within 2 months.  Non-cardiac comorbid conditions present with life expectancy <1 year or that may result in protocol non-compliance (per site investigator’s medical judgment).  Patients who are actively participating in another drug or device investigational study, which have not completed the primary endpoint follow-up period.  Symptomatic heart failure. |
